# Inhibition of KRAS-dependent lung cancer cell growth by deltarasin: blockage of autophagy increases its cytotoxicity

**DOI:** 10.1038/s41419-017-0065-9

**Published:** 2018-02-13

**Authors:** Elaine Lai Han Leung, Lian Xiang Luo, Zhong Qiu Liu, Vincent Kam Wai Wong, Lin Lin Lu, Ying Xie, Ni Zhang, Yuan Qing Qu, Xing Xing Fan, Ying Li, Min Huang, Dai Kai Xiao, Jun Huang, Yan Ling Zhou, Jian Xing He, Jian Ding, Xiao Jun Yao, David C. Ward, Liang Liu

**Affiliations:** 1 State Key Laboratory of Quality Research in Chinese Medicine/Macau Institute For Applied Research in Medicine and Health, Macau University of Science and Technology, Macau, China; 2 Guangzhou Institute of Respiratory Disease, State Key Laboratory of Respiratory Disease, The 1st Affiliated Hospital of Guangzhou Medical College, Guangzhou, China; 3 International Institute for Translational Chinese Medicine, Guangzhou University of Chinese Medicine, Guangzhou, China; 4 State Key Laboratory of Drug Research, Shanghai Institute of Materia Medica, Chinese Academy of Sciences, Shanghai, China

## Abstract

Deltarasin is a recently identified small molecule that can inhibit KRAS–PDEδ interactions by binding to a hydrophobic pocket on PDEδ, resulting in the impairment of cell growth, KRAS activity, and RAS/RAF signaling in human pancreatic ductal adenocarcinoma cell lines. Since KRAS mutations are the most common oncogene mutations in lung adenocarcinomas, implicated in over 30% of all lung cancer cases, we examined the ability of deltarasin to inhibit KRAS-dependent lung cancer cell growth. Here, for the first time, we document that deltarasin produces both apoptosis and autophagy in KRAS-dependent lung cancer cells in vitro and inhibits lung tumor growth in vivo. Deltarasin induces apoptosis by inhibiting the interaction of with PDEδ and its downstream signaling pathways, while it induces autophagy through the AMPK-mTOR signaling pathway. Importantly, the autophagy inhibitor, 3-methyl adenine (3-MA) markedly enhances deltarasin-induced apoptosis via elevation of reactive oxygen species (ROS). In contrast, inhibition of ROS by *N*-acetylcysteine (NAC) significantly attenuated deltarasin-induced cell death. Collectively, these observations suggest that the anti-cancer cell activity of deltarasin can be enhanced by simultaneously blocking “tumor protective” autophagy, but inhibited if combined with an anti-oxidant.

## Introduction

RAS proto-oncogene encoded oncoproteins were classified as the RAS family of small guanosine triphosphate (GTP)-binding proteins and acted as molecular switches by alternating between an active GTP-bound and an inactive GDP-bound form that activate intracellular signaling pathways to control cell proliferation, differentiation, and apoptosis^1,2^. Approximately 20–30% of all human cancers harbor RAS oncogenic mutations, making RAS variants among the most prevalent drivers of cancer^3^. Of the three RAS isoforms, HRAS, NRAS, and KRAS, KRAS is the most frequently mutated RAS isoform (86%) and is commonly found in more than 30% of all lung adenocarcinoma^4^. Moreover, hyperactive KRAS signaling often occurs in common immunological and inflammatory disorders, such as rheumatoid arthritis (RA) and diabetes^5–7^. Effective inhibition of activity may also establish treatments for those diseases. However, inhibiting KRAS signaling has been considered an impossible mission in the past^8^, thus, finding a new approach to inhibit KRAS signaling is extremely important.

The *KRAS* gene is characterized by single base missense mutations, which are predominantly found at codons G12, G13, or Q61^9^. Constitutive activation of KRAS leads to the persistent stimulation of downstream signaling pathways that promote tumorigenesis, including the RAF/MEK/ERK and PI3K/AKT/mTOR signaling cascades^10–13^. Efforts have been made for over three decades to develop effective anti-RAS inhibitors, however, no pharmacological inhibitor of RAS has as yet led to a clinical useful drug^14^. Numerous strategies, including blocking RAS membrane associations, RAS siRNA technology, targeting RAS downstream effector signaling, inhibiting synthetic lethal interactors with mutant RAS, and suppressing cell metabolism are currently being evaluated in preclinical studies^14–18^.

The elucidation of the crystal structure of the cGMP phosphodiesterase 6 delta subunit (PDEδ) protein with a hydrophobic pocket that can interact with a farnesylated hydrphobic cysteine residue at the C terminus of RAS proteins and the identification of deltarasin, a molecule that inhibits the binding of PDEδ to activated RAS proteins, has provided new hope for the development of anti-therapy^19^. Initially, RAS protein undergoes a rapid series of complex post-translational modifications, including permanent C-terminal farnesylation, which ensures it is capable of translocation from endomembranes (EM) to the plasma membrane (PM)^20^, an essential process for KRAS activation function^21^. PDEδ is now regarded as an important chaperone of prenylated small G proteins and a promiscuous prenyl-binding protein of the RAS superfamily, which can bind to RAS protein and recruit it to the PM^21–23^. In particular, PDEδ contains a deep hydrophobic pocket, capable of binding the lipid moiety of farnesyl-acylated proteins such as RAS^24,25^. Therefore, inhibiting the interaction between KRAS/ PDEδ could be a potential therapeutic strategy.

Zimmermann et al.^26^, using a high-throughput screening approach, found one small molecule, deltarasin, that bound the farnesyl-binding pocket of His-tagged PDEδ and disrupted binding to a biotinylated and farnesylated peptide. They also showed that deltarasin inhibits the interaction between KRAS–PDEδ and decreases KRAS binding to the PM in human ductal adenocarcinoma (PDAC) cell lines harboring KRAS gene mutation, resulting in reduction of cell proliferation and induction of apoptosis both in vitro and in vivo. The ability of deltarasin to suppress lung cancer cell growth and the factors affecting deltarasin sensitivity has not yet been explored. Here we show that deltarasin inhibits the growth of lung cancer cell lines, A549, and H358, producing both apoptosis and autophagy, and demonstrate that it also inhibits the growth of A549 cells xenografted into nude mice.

Recent studies have shown that autophagy may be a double-edged sword in relation to cancer^27,28^. On one hand, it can promote tumor cell survival by providing energy for cellular metabolic needs under conditions of nutrient starvation^29^. Alternatively, autophagy can result in progressive consumption of essential cellular components, leading to subsequent cell death. Reactive oxygen species (ROS) have also been identified as signaling molecules that can either promote cell survival or cell death, depending on the cellular contexts and cell types^30,31^. Therefore we have investigated the efficacy of deltarasin in killing KRAS-dependent lung cancer cell lines and the role of autophagy and ROS generation in the cells’ response to deltarasin treatment.

## Results

### Deltarasin induces cytotoxicity and inhibits KRAS–RAF signaling in KRAS-dependent lung cancer cells

**Fig. 1:**
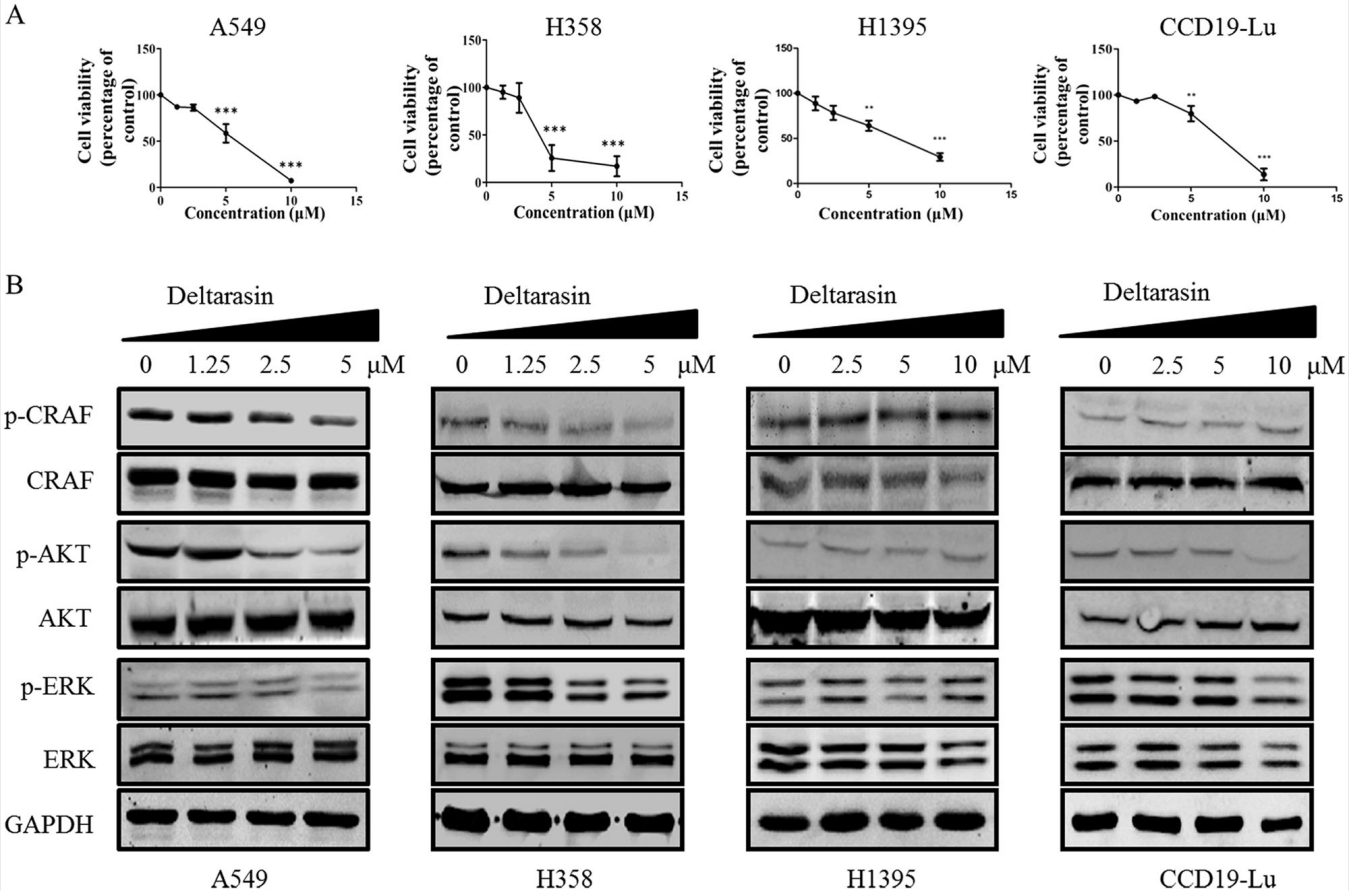
Effect of deltarasin on cell viability and KRAS signaling in KRAS-dependent lung cancer cells, KRAS-independent lung cancer cells with BRAF mutation and WT-KRAS normal lung fibroblast cells . **(A)** A549, H358, H1395, and CCD19-Lu cells were treated with deltarasin (0, 1.25, 2.5, 5, and 10 μM) for 72 h and the percentages of cell viability were measured by MTT assays. **(B)** Representative western blot data of the levels of p-CRAF, CRAF, p-AKT, AKT, p-ERK, ERK, and GAPDH of A549, H358, and CCD19-Lu cells after 24 h deltarasin treatment (0, 1.25, 2.5, and 5 μM). Data were expressed as mean ± SD of three independent experiments (each in triplicate). ***P < 0.001, **P < 0.01 when compared with control

Zimmermann et al.^26^ previously demonstrated the anti-cancer effect of deltarasin on pancreatic cancer cell lines and pancreatic carcinoma with KRAS mutation. We further examined if deltarasin can also induce cytotoxic effects on lung cancer cells with KRAS mutations, since lung cancers occur with much higher frequency than pancreatic cancers in the clinic. A549 and H358 cell lines, which harbor KRAS G12S and G12C point mutations respectively, were used with normal lung fibroblast CCD19-Lu and a BRAF mutation lung cancer cell line, H1395, providing KRAS wild-type (WT) controls. As shown in Fig. 1a, after treatment of deltarasin for 72 h, deltarasin significantly inhibited cell viability in A549 and H358 cells in a dose-dependent manner. The IC_50_ values of these two KRAS-dependent lung cancer cell lines were 5.29 ± 0.07 and 4.21 ± 0.72 μM, respectively. However, the IC_50_ values for the H1395 and CCD19-Lu WT KRAS cell lines were only slightly higher at 6.47 ± 1.63 (H1395) and 6.74 ± 0.57 μM (CCD19-Lu) indicating that deltarasin also exhibits cytoxicity to WT-KRAS control cells. This is not surprising since deltarasin is a benzimidazole that may affect other prenylated proteins in addition to PDEδ, suggesting PDEδ is not the only target of deltarasin. Although deltarasin shows a strong binding affinity to PDEδ, with a *K*_d_ of 38 nM, cytotoxicity could only be demonstrated at micromolar concentrations in both our lung cancer cell lines and previously reported pancreatic cell line studies^26^. This may, in part, reflect bioavailability (e.g., cellular uptake), however a recent publication reports that the KRAS cargo release factor Arl2 induces rapid release of deltarasin from PDEδ, in spite of its high affinity binding^32^.

Active RAS will bind to its downstream effector kinase c-RAF, and subsequently turn on the two downstream growth and anti-apoptotic signaling pathways, the RAF/MEK/ERK and PI3K/AKT signaling cascades^10^. Deltarasin is the first compound reported to block and PDEδ interaction and suppress RAF/MEK/ERK and PI3K/AKT signaling. In Fig. 1b, we further demonstrated that deltarasin can suppress phosphorylation levels of c-RAF, AKT, and ERK in the two lung cancer cell lines. Interestingly, unlike the KRAS mutant lines, deltarasin had little if any effect on the levels of p-CRAF, p-AKT, or p-ERK expression in the WT KRAS cell lines, supporting the contention that deltarasin cytotoxicity to WT KRAS cells may occur via an alternative mechanism.

### Deltarasin induces apoptosis in A549 KRAS-dependent lung cancer cell line

**Fig. 2:**
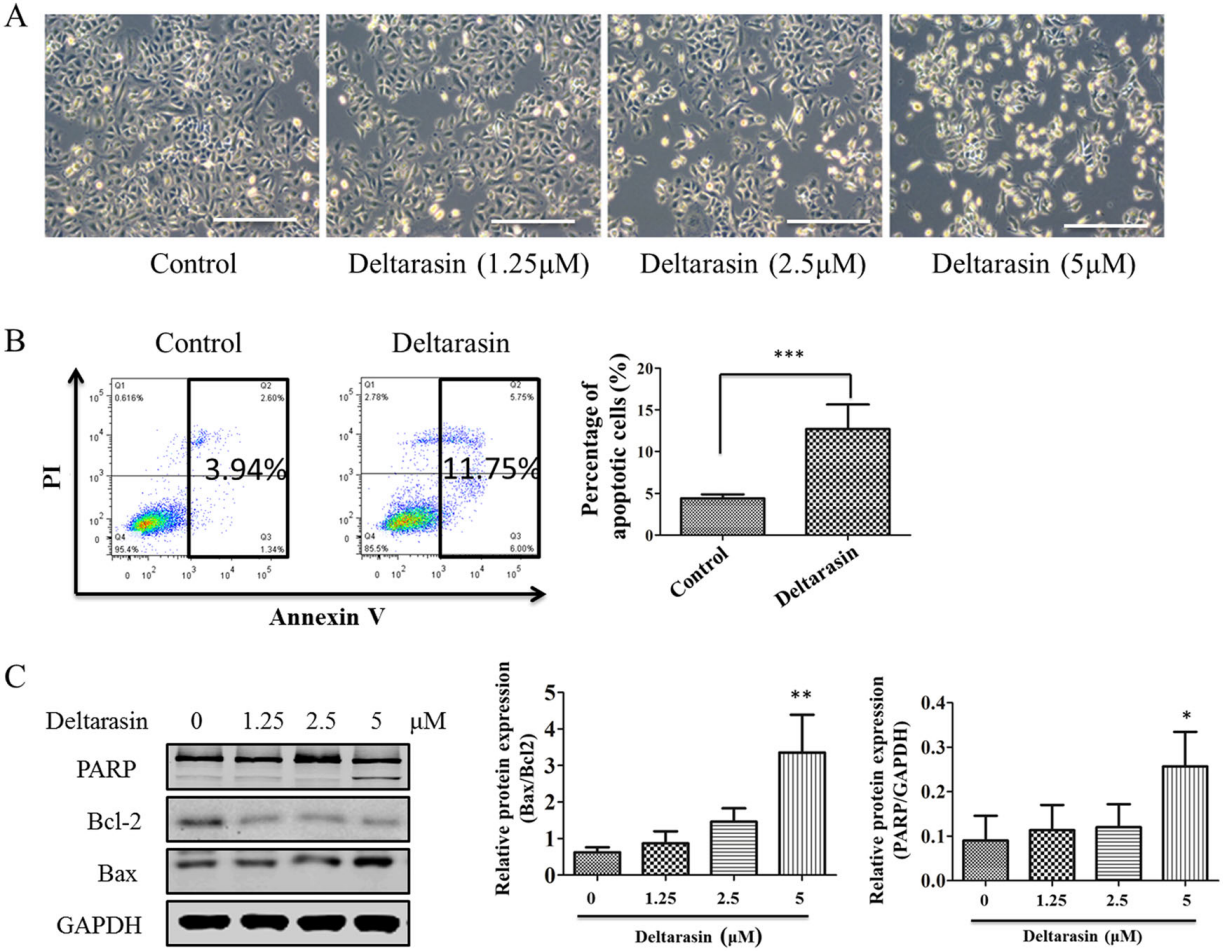
Apoptotic effect of deltarasin in A549 . **(A)** Morphological changes after 24 h exposure to deltarasin (0, 1.25, 2.5, and 5 μM) were captured by an optical microscope with ×100 magnification, scale bar: 100 μm. **(B)** A549 cells were treated with 5 μM deltarasin for 24 h and apoptosis levels were quantitatively measured with flow cytometry after staining cells with Annexin V/ propidium iodide (PI), and triplicate data were plotted as bar chart diagram. **(C)** Representative western blot data of different apoptosis-related protein (PARP, Bcl-2, and Bax) confirmed induction of cellular apoptosis after deltarasin treatment (0, 1.25, 2.5, and 5 μM) for 24 h. Densitometry of the ration of Bax/Bcl2 and cleaved PARP were shown as bar chart. All data were representative of at least three independent experiments and presented as mean ± SD, *P < 0.05, **P < 0.01, ***P < 0.001 compared with control

Using A549 cells as a representative lung cancer cell line with KRAS mutations, we next determined whether the observed growth inhibition was due to apoptosis or necrosis. As illustrated in Fig. 2a, after treatment with deltarasin, cell morphology of A549 is altered in a concentration-dependent manner, a proportion of A549 cells detached from the culture dish and rounded up, which are predictive apoptotic features. Next, applying standard Annexin V-FITC/PI staining followed by flow cytometry analysis, the percentage of apoptotic and necrotic cells were quantitatively measured. Results showed that deltarasin significantly induced apoptosis in A549 cells when compared with the untreated cells (Fig. 2b). Furthermore, to examine deltarasin-induced apoptosis in A549 cells, the expression levels of several well-characterized apoptotic proteins were analyzed by western blotting. Results showed that an increase in the expression of the pro-apoptotic protein Bax and a reduction of expression of anti-apoptotic Bcl-2 were observed in the deltarasin-treated cells. In addition, deltarasin treatment resulted in induction of the cleavage of PARP at 5 μM (Fig. 2c), also consistent with apoptosis. The densitometry quantitation of the ratio of Bax/Bcl2 protein expression and PARP cleavage relative to GAPHD is shown as a bar chart in Fig. 2c. Taken together, these results revealed that deltarasin induces apoptosis in lung cancer cells, as was previously shown with pancreatic cancer cells.

### Deltarasin suppresses tumor growth in A549 xenograft

**Fig. 3:**
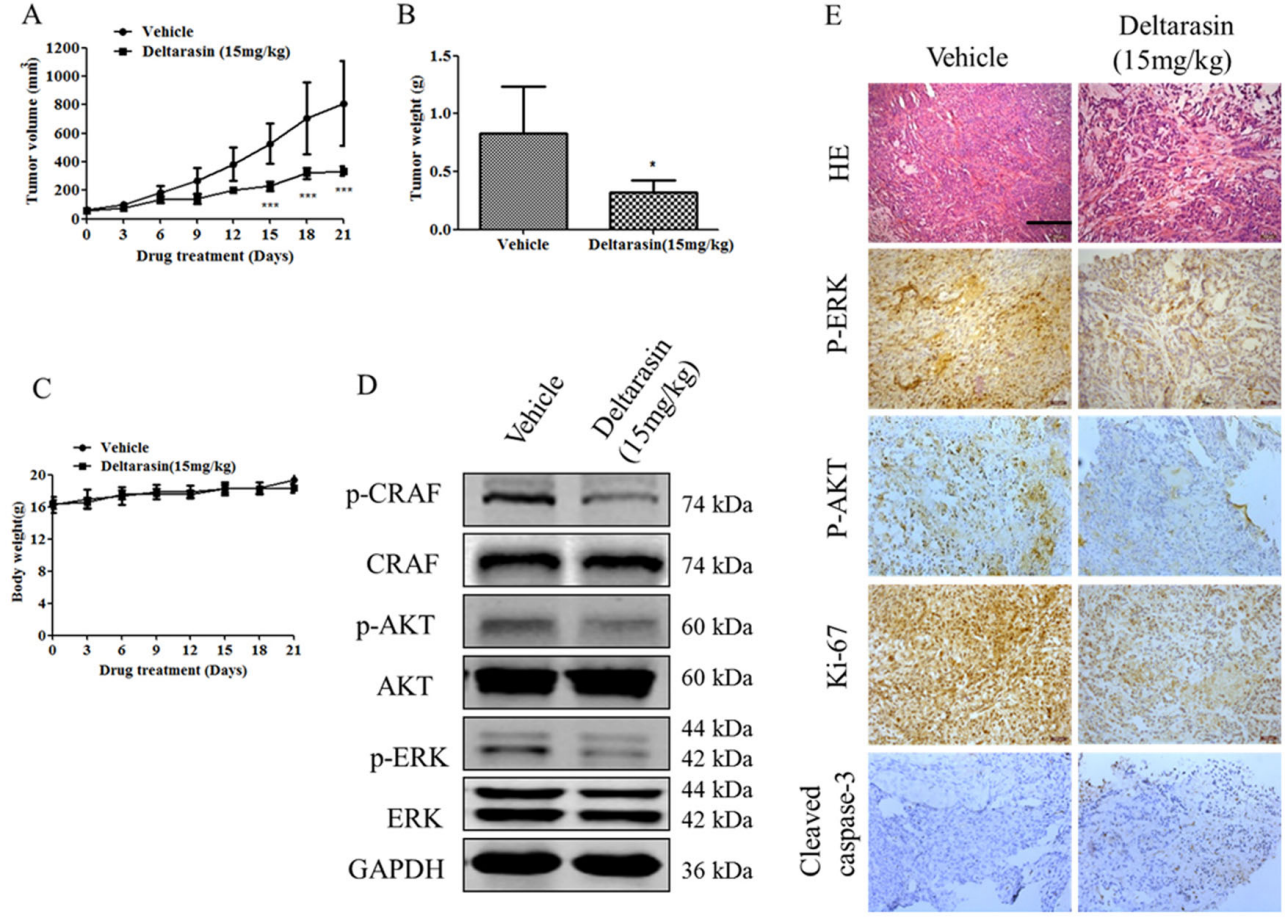
Tumor suppression effect of deltarasin in A549 xenograft . **(A)** Tumor growth curve was shown with deltarasin i.p. treatment regimen lasting for duration of 21 days. ***P < 0.001 vs. vehicle-treated group. Data represent mean ± SD (*n* = 5). **(B)** The average tumor weight of control group and the deltarasin-treated tumor at the day of harvest (day 21). **(C)** Quantification of body weight mice for each group. Data are presented as mean ± SD; *n* = 5 per group. **(D)** Immunoblot analysis of ERK, p-ERK, AKT, p-AKT, CRAF, and p-CRAF in the protein lysates prepared from vehicle-treated tumors and deltarasin-treated tumors. GAPDH was used as a loading control. **(E)** Images represent hematoxylin & eosin (H&E) and immunohistochemical staining for p-ERK, p-AKT, Ki-67, and cleaved caspase-3 antibody, scale bar, 100 μm

We next assessed the in vivo efficacy of deltarasin in the context of mutant KRAS. A549 cells were injected into nude mice, and the tumors were allowed to grow to about 60 mm^3^ in size and treated daily with deltarasin for 21 days. Figure 3a shows that deltarasin suppressed tumor growth starting at day 9 and showed significant suppression from day 15 to day 21 when compared to the vehicle-treated controls. At the day of harvest, the net tumor mass was determined and the average tumor weight of the deltarasin treatment group was 57% less than the average tumor weight of the control group (Fig. 3b). The mice (*n* = 5) exhibited no significant body weight loss or apparent toxicity after treatment with deltarasin (Fig. 3c). We further examined the effect of deltarasin treatment on KRAS-mediated RAF, MEK/ERK, and PI3K/AKT cascades in protein extracts derived from vehicle- and deltarasin-treated tumors. Consistent with the in vitro data, we observed a significant suppression of CRAF, ERK, and AKT phosphorylation (Fig. 3d). Immunohistochemical analysis also showed that treatment with deltarasin decreased levels of ERK and AKT phosphorylation (Fig. 3e), indicating that the growth inhibition induced by deltarasin is associated with suppression of KRAS-mediated signaling. Moreover, immunohistochemistry analysis of the tumors from deltarasin-treated mice showed a large reduction in cell proliferation as indicated by Ki-67 staining and a prominent increase of apoptotic cells as indicated by cleaved caspase-3 staining (Fig. 3e). Taken together, these data demonstrated that deltarasin is effective in suppressing KRAS-driven lung tumor growth.

### Deltarasin inhibits the interaction of KRAS with PDEδ and its downstream signaling pathway

**Fig. 4:**
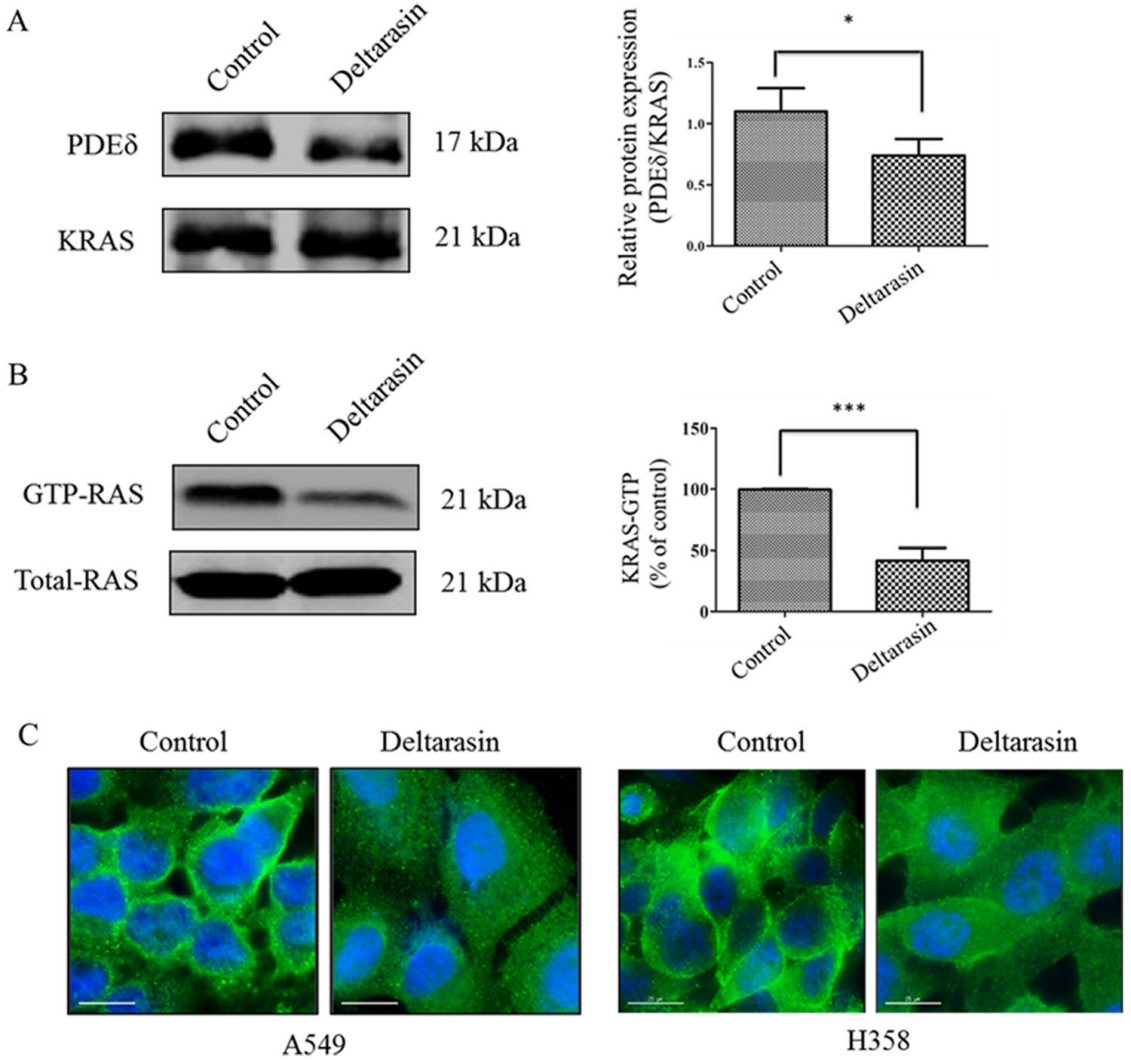
Deltarasin inhibits the binding of GTP to Ras and interaction of KRAS with PDEδ . **(A)** Co-immuoprecipitation of PDEδ and KRAS using PDEδ and monoclonal antibodies for the control and deltarasin treatment group in H358 cell line. **(B)** KRAS–GTP levels were determined by incubating the protein lysates from A549-treated 5 μM deltarasin for 24 h with glutathione S-transferase (GST)-tagged Ras binding domain (RBD) immobilized on glutathione beads. Percentage of KRAS–GTP bindings were compared with untreated control. **(C)** Cellular localization of was observed by fluorescence microscopy after 5 μM deltarasin treatment for 24 h in both A549 and H358 cells (green signal: immunofluorescence signal of primary antibody against KRAS; Blue signal: Hoechst staining on cell nucleus. Magnification: ×40; scale bar: 15 μm). The results were expressed as the mean ± SD of three independent experiments, *P < 0.05; ***P < 0.001 vs. control

In Fig. 4a, coimmunoprecipitation studies using KRAS antibodies showed that deltarasin treatment reduces the amount of PDEδ in the immunoprecipitate, demonstrating that deltarasin inhibits -PDEδ interactions in the H358 cell line used. We next treated A549 cells with deltarasin and performed the RAS activation assay to measure the level of GTP-RAS. As shown in Fig. 4b, treatment of A549 cells with deltarasin significantly decreases the amount of GTP-RAS observed relative to the control cells. Finally, as shown in Fig. 4c, similar to the results previously reported in pancreatic cancer cells^26^, treatment of A549 cells and H358 cells with deltarasin also significantly reduces the amount of protein at the PM and displaced KRAS into the EM. In contrast, in the control untreated cells, it was mainly localized at the PM. The above observations demonstrate that deltarasin can inhibit the interaction of KRAS with PDEδ and suppresses the RAS downstream signaling pathways in lung cancer cells.

### Deltarasin induces autophagy in lung cancer cells

**Fig. 5:**
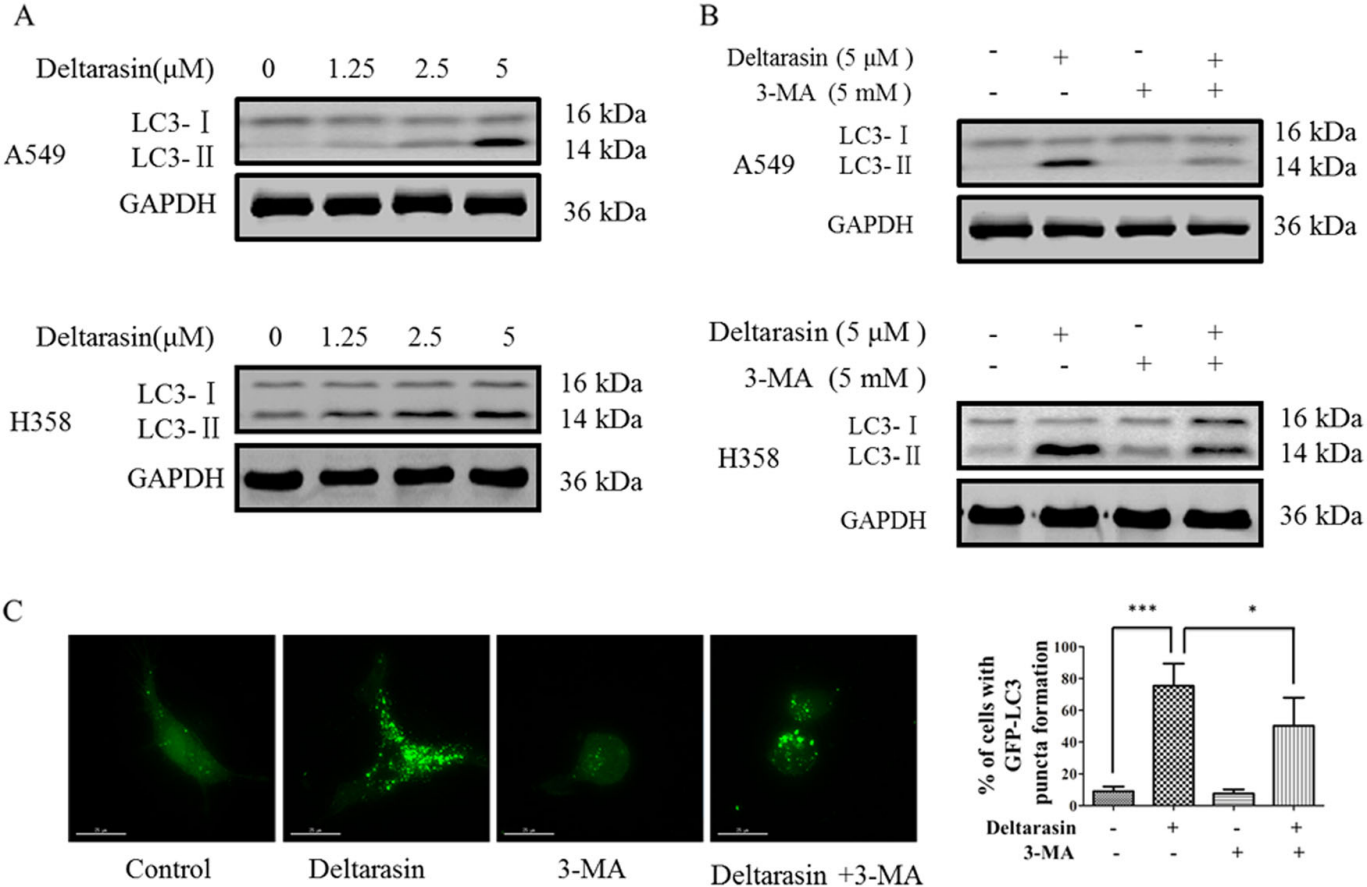
Autophagy was induced by deltarasin and inhibited by 3-MA in lung cancer cells . **(A)** A549 and H358 cells were treated with deltarasin (0, 1.25, 2.5, 5 μM) for 24 h. The conversion of LC3-I to LC3-II was determined by western blot with GAPDH as a loading control. Representative western blot data were shown. **(B)** A549 and H358 cells were treated with vehicle, 5 μM deltarasin, 5 mM 3-MA, or a combination of both deltarasin and 3-MA for 24 h, and expression of LC3 and GAPDH was analyzed by western blot. **(C)** A549 cells were transfected with GFP-LC3 plasmid for 24 h, and then treated with vehicle, 5 μM deltarasin, 5 mM 3-MA, or in combination of both for 24 h. After treatment, deltarasin-induced autophagy manifesting as fluorescence green GFP-LC3 puncta was assessed by fluorescence microscopy, magnification: ×60, scale bar: 15 μm. The percentage of cells with increased GFP-LC3 puncta formation was represented as a bar chart. The results were expressed as the mean ± SD of three independent experiments, ***P < 0.001 vs. control; *P < 0.05 vs. deltarasin-treated alone group

Zimmerman et al.^26^ did not explore whether autophagy played a role in the deltarasin-mediated cell death of pancreatic cancer cells. In this study, we demonstrate that deltarasin-treated lung cancer cells induce “tumor cell-protective” autophagy as well as apoptosis. The conversion of soluble LC3-I to lipid-bound LC3-II is associated with autophagosome formation, which can be used as a marker for autophagy induction^33^. As illustrated in Fig. 5a, treatment with deltarasin distinctly facilitated the conversion of LC3-I to LC3-II in A549 and H358 cells in a dose-dependent manner. In contrast, the accumulation of LC3-II induced by deltarasin could be suppressed in the presence of autophagy inhibitor, 3-methyl adenine (3-MA), a classic type III PI3K inhibitor in both lung cancer cell lines (Fig. 5b). Similarly, we evaluated the autophagy activity of deltarasin by transiently expressing the green fluorescent protein microtubule-associated protein light chain 3 (GFP-LC3) in A549 cells. As indicated in Fig. 5c, upon deltarasin treatment, an increased level of GFP-LC3 puncta was observed relative to the untreated control cells, suggesting autophagosome formation was induced by deltarasin, while the number of GFP-LC3 puncta was significantly diminished in the presence of autophagy inhibitor 3-MA. Taken together, these data demonstrated for the first time that deltarasin induced autophagy in both lung cancer cell lines.

### Deltarasin induces autophagy through AMPK-mTOR-dependent pathway

**Fig. 6:**
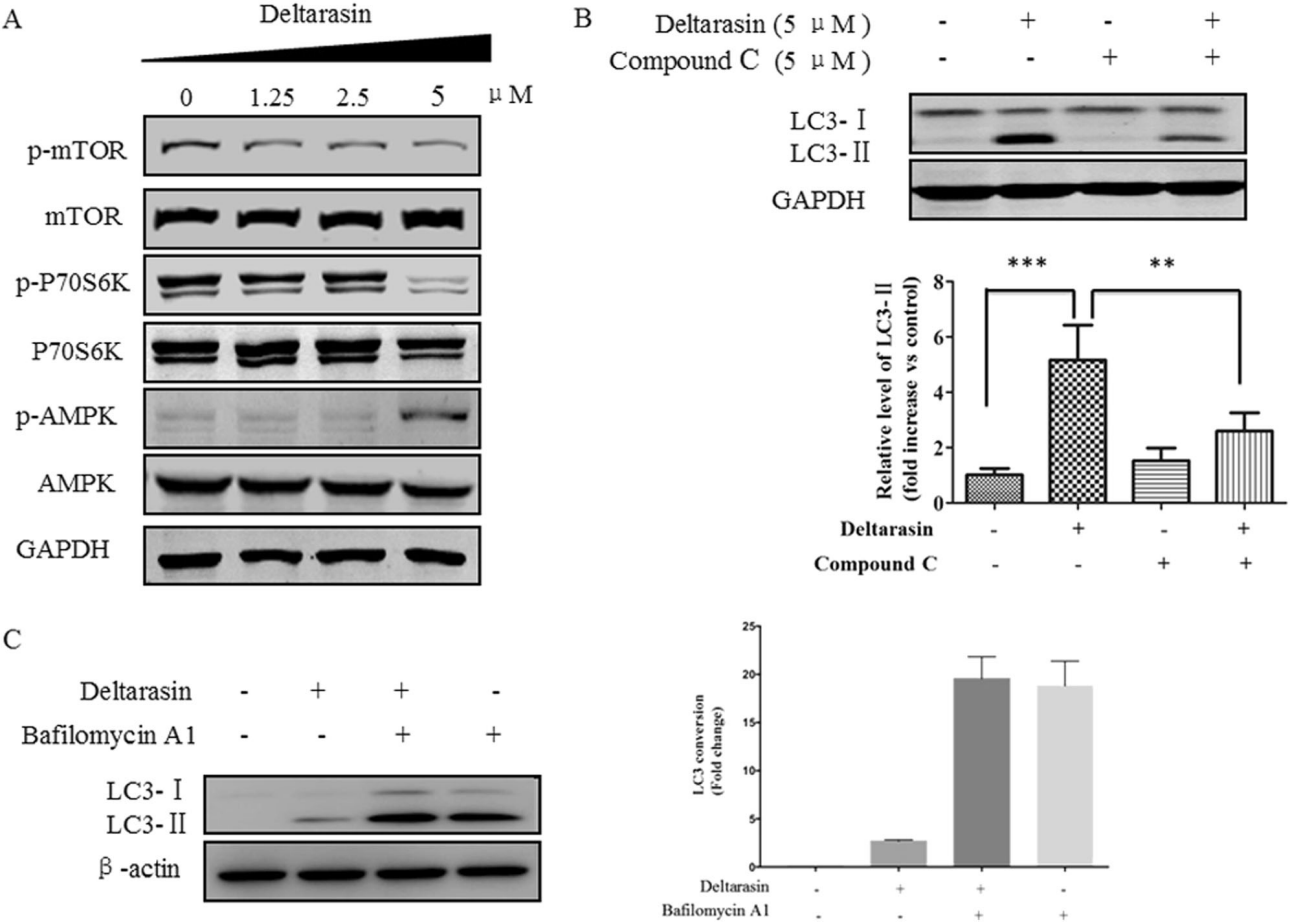
Deltarasin induced autophagy through the activation of AMPK-mTOR pathway in A549 cells . **(A)** A549 cells were treated with 0, 1.25, 2.5, and 5 μM deltarasin for 24 h and the levels of p-mTOR, mTOR, p-P70S6K, P70S6K, p-AMPK, AMPK, and GAPDH were evaluated by western blot. **(B)** Cells were treated with vehicle, 5 μM deltarasin, 5 μM Compound C, or a combination of both for 24 h, then the relative levels of LC3 were analyzed by western blot with densitometry. Representative western blot data were shown. The results were expressed as the mean ± SD of three independent experiments. ***P < 0.001 when compared with control and **P < 0.01 when compared with the deltarasin-treated alone group. **(C)** A549 cells were treated with 5 μM of deltarasin in the presence or absence of lysosomal protease inhibitors (50 nM) bafilomycin A for 24 h. Cell lysates were analyzed by western blot for LC3 conversion. Data were expressed as a fold change relative to the DMSO-treated negative control. Bar charts were representatives of three independent experiments

To further examine the molecular mechanisms of deltarasin-induced autophagy, we determined the possible involvement of AMP-activated protein kinase (AMPK)-mammalian target of rapamycin (mTOR) signaling pathway. mTOR, which is a member of the phosphatidylinositol 3-kinase (PI3K) cell survival pathway, and plays an important role in the regulation of cell growth and proliferation by monitoring nutrient availability, cellular energy levels, oxygen levels, and mitogenic signals^34^. AMPK, which is a key energy sensor and regulates cellular metabolism to maintain energy homeostasis, can promote autophagy. It was further reported that mTOR is a sensor of changes in the cellular energy state via AMPK^35^. Activation of this protein kinase inhibits mTOR-dependent signaling and inhibits protein synthesis. As shown in Fig. 6a, using A549 as representative cells, treatment of A549 with deltarasin suppressed mTOR and p70S6K phosphorylation, with concomitant upregulation of phospho-AMPK. In addition, accumulation of LC3-II was suppressed when co-treated deltarasin with AMPK inhibitor compound C (Fig. 6b). This results suggest that the deltarasin-induced autophagy in A549 cells is mediated through the activation of the AMPK-mTOR signaling pathway. Induction of autophagy is indicated by an increased formation of GFP-LC3 puncta as observed by fluorescence microscopy, or LC3 lipidation using western blot, can be resulted from either from an induction of autophagic flux or failure in fusion of autophagosomes and lysosomes. Hence, we measured the conversion of soluble LC3-I to lipid-bound LC3-II in the presence of lysosomal protease inhibitors (bafilomycin A). As expected, deltarasin significantly increased the rate of LC3-II formation in the presence of bafilomycin A when compared with the bafilomycin A or deltarasin alone groups (Fig. 6c). This result suggested that deltarasin induced autophagic activity through enhanced autophagic flux and autophagosome formation.

### Inhibition of deltarasin-induced autophagy enhances cell death of lung cancer cells

**Fig. 7:**
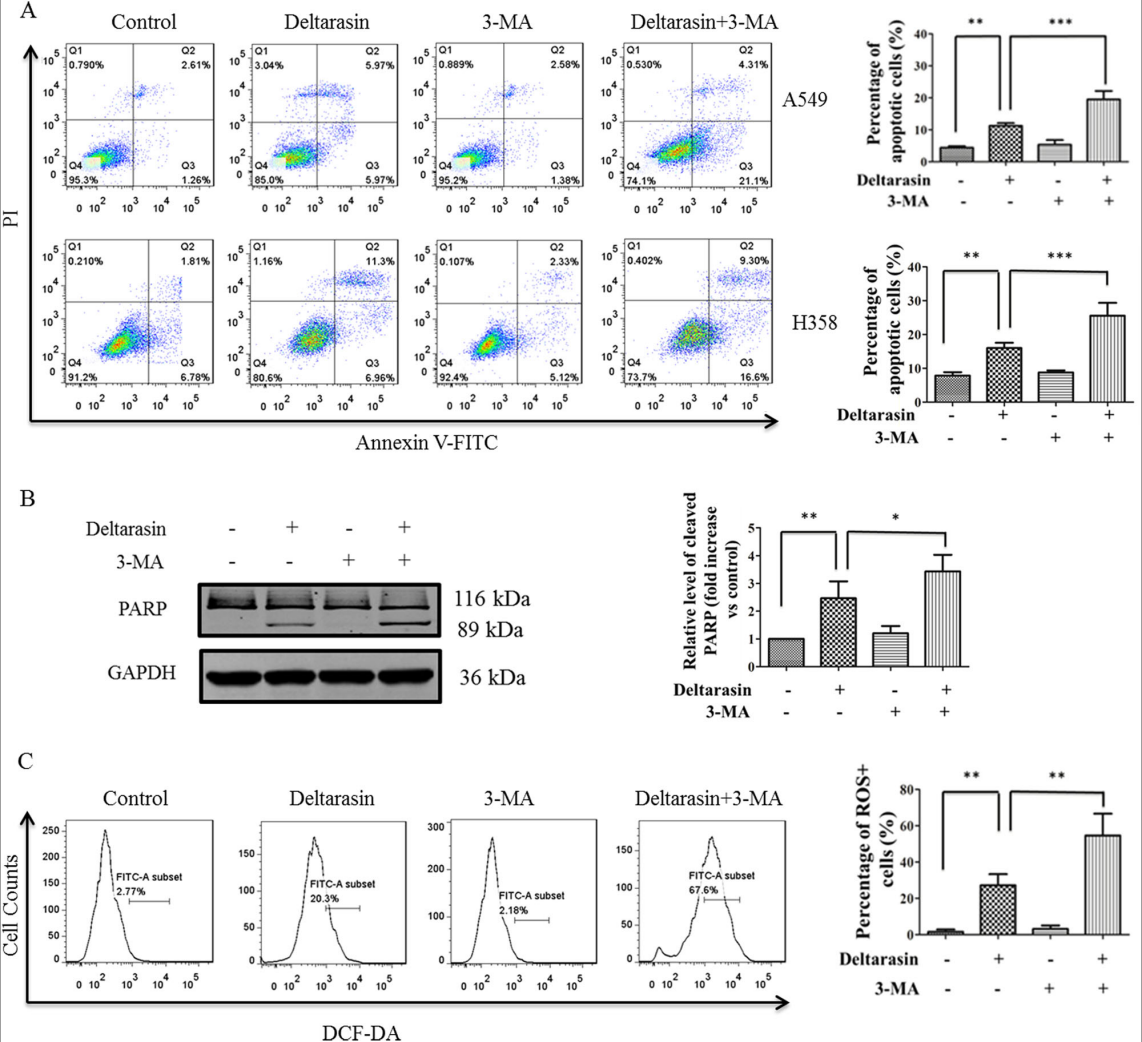
Inhibition of autophagy enhanced the anti-cancer effect of deltarasin in KRAS-dependent lung cancer cells . **(A)** Both A549 and H358 cells were treated with vehicle, 5 μM deltarasin, 5 mM 3-MA, or a combination of both for 24 h, and cell apoptosis was measured by Annexin V/PI double staining with flow cytometry. Bar chart diagram of the levels of apoptosis of three representative experiments are presented. **(B)** A549 cells were treated with vehicle, 5 μM deltarasin, 5 mM 3-MA, or a combination of both for 24 h, and the levels of PARP cleavage were analyzed by western blot. Bar chart diagram of the densitometry quantitative analysis of three representative western blots are presented. **(C)** A549 cells were treated with 5 μM deltarasin, 5 mM 3-MA, or a combination of both for 24 h, then ROS generation was measured by flow cytometer after DCF-DA staining. The results were expressed as the mean ± SD of three independent experiments, **P < 0.01 when compared with control and ***P < 0.001 or *P < 0.05 when compared with the deltarasin-treated alone group

Low levels of autophagy may protect cells from stress and cell death, and autophagy induction promotes tumor resistance to chemotherapy^36,37^. Thus, autophagy inhibition could be used in combination with chemotherapy to increase the sensitivity of cancer cells to drugs. Therefore, we further investigated the mechanism of deltarasin-induced autophagy on both A549 and H358 cells and determined the role of autophagy on deltarasin sensitivity. As shown in Fig. 7a, treatment of A549 and H358 cells with 5 μM deltarasin for 24 h induced 11.25% and 15.99% of cell apoptosis, respectively; however, when cells were co-treated with deltarasin and 3-MA, it resulted in 21.7% and 25.54% of cell apoptosis, respectively, indicating deltarasin-induced autophagy is tumor-protective which should be blocked in order to enhance the anti-cancer effect of deltarasin.

Moreover, the inhibition of autophagy further promoted the cleavage of PARP when compared with the deltarasin alone treatment group (Fig. 7b). In addition, as shown by the enhanced levels of DCF-DA staining, a measure of ROS production, inhibition of autophagy further increased ROS generation (Fig. 7c), leading to further cell damage, suggesting that deltarasin evoked protective autophagy. Taken together, these data demonstrated that inhibition of deltarasin-induced autophagy potentiated apoptosis of A549 cells.

### Blocking deltarasin-induced ROS suppresses autophagy and apoptosis

**Fig. 8:**
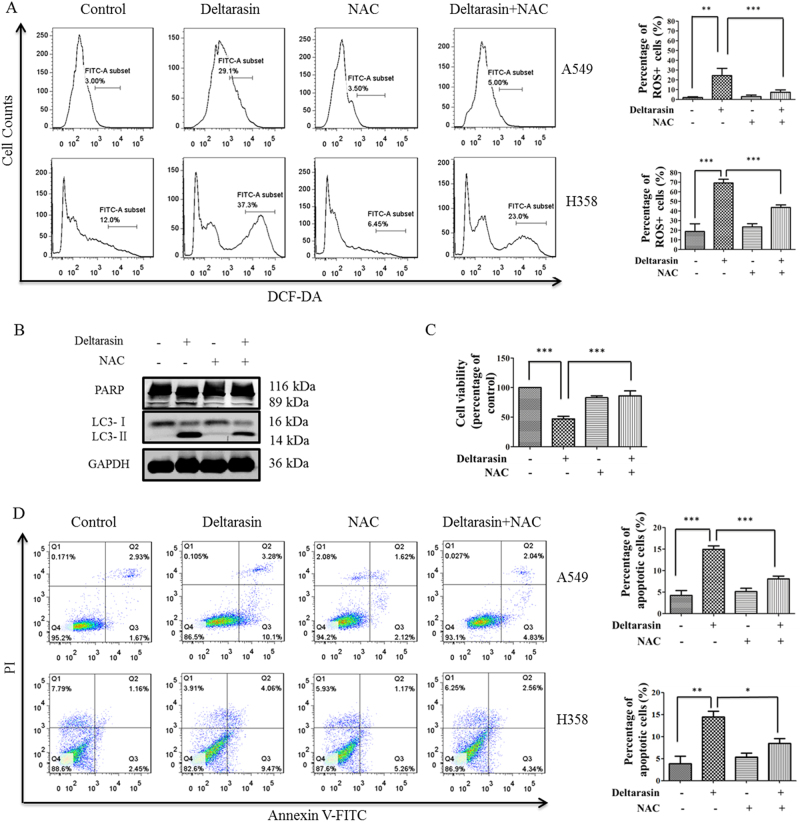
Deltarasin induced the accumulation of ROS and NAC reversed deltarasin-induced apoptosis . **(A)** A549 and H358 cells were treated with vehicle, 5 μM deltarasin, 5 mM NAC, or in combination of both for 24 h, then cells were stained with DCF-DA and the levels of ROS were analyzed by flow cytometry. **(B)** A549 cells were treated with vehicle, 5 μM deltarasin, 5 mM NAC, or in combination of both for 24 h and the levels of PARP cleavage and LC3 were evaluated by western blot. **(C)** A549 cells were treated with vehicle, 5 μM deltarasin, 5 mM NAC, or in combination of both for 24 h and the percentage of cell viability was measured by MTT assays. **(D)** A549 and H358 cells were treated with vehicle, 5 μM deltarasin, 5 mM NAC, or in combination of both for 24 h, and the levels of cell apoptosis were measured by Annexin V/PI double staining with flow cytometry. The results were expressed as the mean ± SD of three independent experiments, **P < 0.01, ***P < 0.001, when compared with control or deltarasin-treated alone group

The role of ROS in cancer is controversial: low levels of ROS can support growth of cancer, however, high levels of ROS can trigger oxidative stress and protein damage of cancer cells, resulting in apoptosis^38,39^. Similarly, autophagy was reported to have dual roles in cancer regulation, and it was reported that ROS can induce autophagy^40^, therefore, we further examined whether deltarasin-induced autophagy is dependent on ROS generation in lung cancer cells, and further explored the role of ROS in deltarasin-induced apoptosis and autophagy. As shown in Fig. 8a, deltarasin significantly elevated intracellular ROS levels, whereas a general ROS scavenger, *N*-acetyl cysteine (NAC), suppressed the high level of ROS induced by deltarasin in both cancer cell lines.

Notably, scavenging ROS with NAC suppressed both PARP cleavage, and the accumulation of LC3-II in A549 cells, leading to blocking of autophagy (Fig. 8b). However, flow cytometry analysis showed that co-treatment with NAC decreased deltarasin-induced cell cytotoxicity and apoptosis in A549 and H358 cells (Fig. 8c, d). All these data suggested that although there is beneficial anti-cancer effect of blocking autophagy, simultaneously, blocking ROS will weaken its beneficial effect due to suppressing its oxidative damage to cancer cells. Using direct autophagy inhibitor (e.g., 3-MA), rather than an anti-oxidant (e.g., NAC), would appear to be highly desirable to enhance the anti-cancer effect of deltarasin in the future clinical application.

## Discussion

**Fig. 9:**
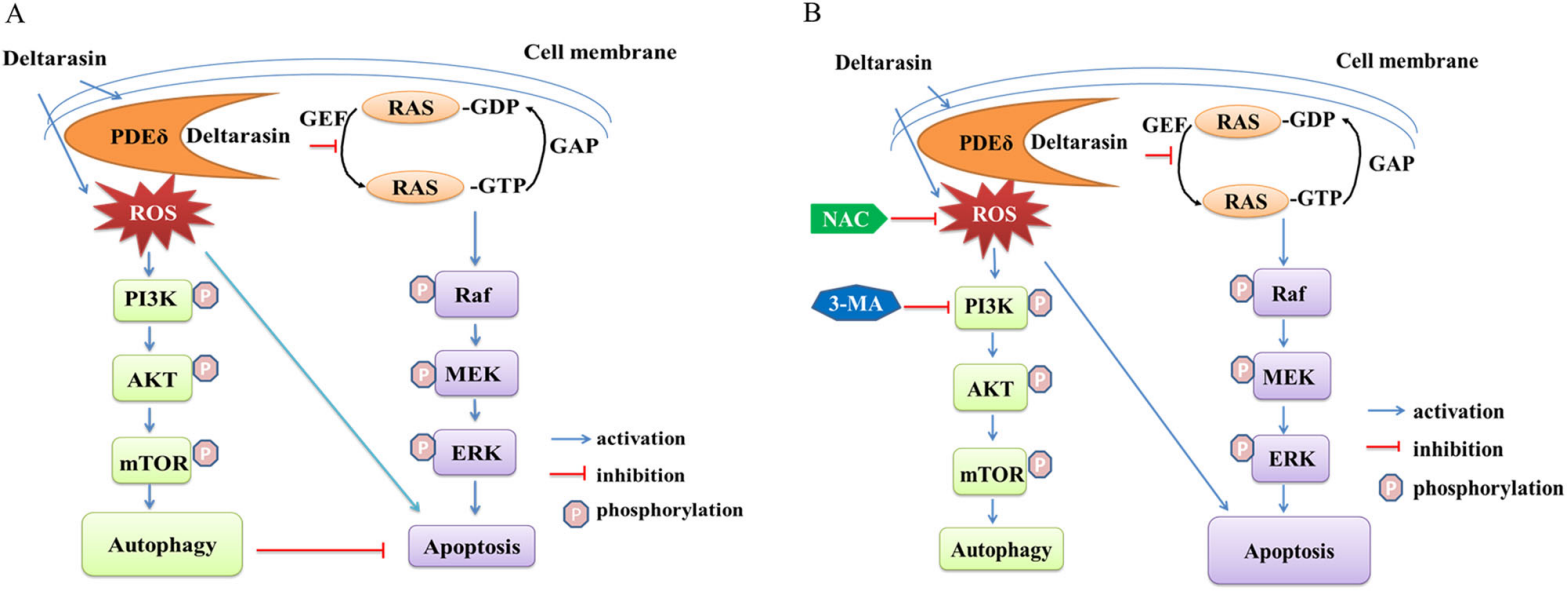
Hypothetical mechanism for deltarasin-induced autophagy and apoptosis in KRAS-dependent lung cancer cells . Deltarasin induces apoptosis and pro-survival autophagy mediated by triggering ROS accumulation. **(A)** In the absence of 3-MA and NAC. **(B)** In the presence of 3-MA and NAC

Recent studies^25,26^ showed that deltarasin inhibited -PDEδ interactions by selectively binding to the prenyl-binding pocket of PDEδ with nanomolar affinity, thus suppressing oncogenic RAS/RAF signaling and inhibiting in vitro and in vivo growth of human pancreatic ductal adenocarcinoma cells that is harboring KRAS mutations. These observations open a new horizons for the treatment of other KRAS-associated clinical disorders, since KRAS was considered as an undruggable target in the last decade^14^. In fact, KRAS mutations are the most common oncogenic mutation in lung adenocarcinoma and KRAS hyperactivation is highly associated with many complex immunological and inflammatory disorders, such as RA and diabetes, which are difficult to cure and drug resistance is common^41^. To our knowledge, this is the first report evaluating the anti-cancer effect of deltarasin in lung cancer cells or in vivo lung tumors, and the factors affecting deltarasin sensitivity. We demonstrate that deltarasin inhibits the interaction of with PDEδ as well as the downstream RAF/MEK/ERK, PI3K/AKT signaling pathways in KRAS-dependent lung cancer cells. Moreover, we demonstrate that deltarasin also suppressed lung cancer cell growth, both in vitro and in vivo. In addition, we have demonstrated that deltarasin can increase intracellular ROS levels and induce autophagy in lung cancer cells, and we found that autophagy plays a protective role in the process, which weaken the overall anti-cancer effect of deltarasin (Fig. 9). Thus, blocking autophagy can sensitize cells to deltarasin treatment.

Autophagy plays a role in the catabolic pathway that cells use to support metabolism in response to starvation and to clear damaged proteins and organelles in response to stress^42^. It has been recently reported that autophagy is an important mechanism for sustaining glycolytic RAS-mediated oncogenic transformation and KRAS oncogene upregulates basal autophagy to meet tumor cell survival in starvation and tumorigenesis^43,44^. Autophagy is a cellular process that engulfs damaged organelles and cytoplasmic material in double membrane vesicles, which later fuse with lysosomes for degradation and recycling of their content^45^. During autophagy, protein aggregates or organelles are sequestered in autophagosomes and degraded in lysosomes to provide recycled building blocks for anabolism and energetics, lipidated LC3-II, used as a marker for autophagy, is tightly associated with the autophagosomes^46^. In our study, we found that deltarasin treatment caused the conversion of LC3-I to LC3-II in both KRAS-dependent lung cancer cells, as shown by the increases of GFP-LC3 puncta cells. This phenomenon was reversed by the autophagy inhibitor 3-MA, suggesting that deltarasin-induced autophagy in A549 cells. Furthermore, our results revealed that deltarasin-induced autophagy is dependent on the AMPK-mTOR signaling pathway. In cancer therapy, autophagy seems to play a dual role, either promoting or inhibiting apoptosis in cancer cell. To determine whether deltarasin-induced autophagy plays a pro-survival or pro-death effect in KRAS-dependent cancer, pre-treatment with autophagy inhibitor 3-MA enhanced deltarasin-mediated cytotoxicity, suggesting that deltarasin-induced autophagy may play a protective role in KRAS-dependent lung cancer cells. In addition, autophagy maintains functioning mitochondria to support KRASG12D-driven NSCLC tumor metabolism, growth, and fate^47^. Apoptosis-stimulating of p53 protein 2 (ASPP2) inhibits autophagy to enhance cellular senescence, and inhibit tumor growth, which is consistent with our findings^48^.

ROS has been proposed to both promote or delay cancer cell initiation and expansion, and it plays a significant role in physiological and pathological processes^31^. These conflicting outcomes may be explained by multiple mechanisms and may depend on cancer types or types of oncogenic mutations^49^. Recently, emerging evidence has indicated that ROS generation is a stimulus to trigger cell apoptosis and autophagy. ROS-mediated apoptosis and autophagy have been observed in various cancer cells types, where autophagy regulation and ROS production are strongly inter-connected. In addition, low intracellular ROS levels may contribute to oncogenic KRAS-mediated PDAC formation, and Nrf2 anti-oxidant program can promote KRASG12D-initiated pancreatic and lung proliferation and tumorigenesis^38,50^. Thus, clarification of the role of ROS in regulating deltarasin treatment effect is pivotally important.

Our results showed that deltarasin significantly triggered ROS generation, and the accumulation of ROS could be significantly blocked by the ROS scavenger, NAC. Then we further focused on whether ROS production was associated with deltarasin-induced apoptosis and autophagy. Interestingly, the level of deltarasin-induced apoptosis was significantly decreased after pre-treatment with NAC, although at the same time, autophagy was inhibited. Furthermore, deltarasin-induced cleavage of PARP was reversed by NAC suggesting that deltarasin-induced cell apoptosis was dependent on ROS generation. Meanwhile, in the presence of NAC, the LC3-II conversion that was induced by deltarasin decreased, suggesting that ROS plays a vital role in deltarasin-induced apoptosis. Overall, it seems that the beneficial effect of blocking autophagy is masked by the loss of apoptosis induced by ROS due to ROS inhibition by NAC. Collectively, these data implied that combination treatment of deltarasin with an anti-oxidant is not beneficial to anti-cancer treatment.

In summary, our results revealed that deltarasin was effective in inducing apoptosis and pro-survival autophagy mediated by increasing ROS production, and that autophagy played a protective role which increased cell resistant to deltarasin. Thus, we speculate that the application of a direct autophagy inhibitor combined with deltarasin could provide a better tailored combinational treatment strategy to improve the efficiency and safety of deltarasin. Since ROS plays an essential role in deltarasin-induced cytotoxicity, therefore anti-oxidant therapeutics increases drug resistance to deltarasin. These observations should be taken into consideration when using this PDEδ inhibitor in clinical studies in treating cancer and inflammatory diseases in the future. Finally, while it is apparent that disrupting KRAS–PDEδ interactions is a viable approach to inhibiting mutant KRAS-driven tumor cell growth, it is also apparent that additional compounds with greater target selectivity, as well as lower cytotoxicity and higher binding affinity, need to be identified. Such studies are currently underway.

## Materials and methods

### Chemical reagents and antibodies

Deltarasin, bafilomycin A, and 3-MA were purchased from Selleck Chemicals Co. Ltd, compound C was purchased from Sigma-Aldrich, and they were dissolved in dimethyl sulfoxide (DMSO) to a 10 or 50 mM stock concentration and stored as small aliquots at −20° C until further use. GAPDH, β-actin, C-Raf, p-C-Raf, p-AKT (Ser473), p-ERK (Thr202/Thy204), ERK, PARP, Bax, Bcl-2, p-p70s6k, p70s6k, p-AMPK, AMPK, LC3 antibodies were purchased from Cell Signaling Technology. Anti-AKT, Kras antibody was purchased from Santa Cruz Biotechnology.

### Cell lines and cell cultures

All cell lines were obtained from the American Type Culture Collection (ATCC). Human NSCLC cell lines (H358, A549, and H1395) were cultured in RPMI 1640 supplemented with 10% FBS. CCD19-Lu cells were grown in MEM medium supplemented with 10% FBS. All cell lines were incubated at 37° C and were maintained in an atmosphere containing 5% CO_2_.

### Cell viability assay

About 3000 cells were seeded in 96-well plates and cultured overnight for cell adhesion, and treated with DMSO or various concentrations of deltarasin for 72 h. At the end of the incubation, 10 μl of MTT (5 mg/ml, Sigma) was added into each well for 4 h at 37° C, then the crystals were dissolved in 100 μl of the resolved solution (10% SDS and 0.1 mM HCl). The absorbance at 570 nm was measured using a microplate reader (Tecan, Morrisville, NC, USA). The cell viability was calculated relative to untreated controls. At least three independent experiments were performed and the data were plotted as curve graph.

### Cell apoptosis assays

Apoptosis was measured using the annexin V-FITC apoptosis detection kit (BD Biosciences, San Jose, CA, USA), according to the manufacturer’s protocol. Briefly, A549, H358, and H1395 cells (1.0 × 10^5^ cells/well) were allowed to attach in a six-well plate for 24 h, cells were treated with deltarasin for 24 h. Subsequently, cells were trypsinized, washed with PBS, and stained with 100 μl binding buffer containing 2 μl annexin V FITC (2.5 μg/ml) and 5 μl propidine iodide (PI) (50 μg/ml) incubated in the dark at room temperature for 15 min, before further addition of 400 μl of 1× Annexin-binding buffer. The stained cells were analyzed quantitatively using a BD Aria III Flow Cytometer (BD Biosciences, San Jose, California, USA). Data were analyzed by Flow Jo software.

### Transient transfection and detection of autophagy

GFP-LC3 expression vector was utilized to monitor and quantify the induction of autophagy following the 3rd autophagy guidelines^48^. Briefly, A549 cells were seeded at a density of 2 × 10^5^ cells/well in six-well plates, according to the manufacturer’s instructions. Lipofectamine 2000 was incubated with GFP-LC3 in Opti-MEM-reduced serum medium for 20 min at room temperature. The mixture was added drop by drop to the cells and then incubated for 4–6 h. The DNA/Lipofectamine 2000 medium was replaced by fresh medium and cultured for another 24 h. Then, 5 μM deltarasin or 5 mM 3-MA was added to the cells and after the end of the treatment period, autophagy was measured by counting the increased percentage of cells with punctate GFP-LC3 fluorescence using API Delta Vision Live-cell Imaging System (Applied Precision Inc., GE Healthcare Company, Washington, USA)^51^. The percentage of autophagic cells was calculated by counting the number of the cells showing increased punctuate pattern of LC3 fluorescence (≥10 dots/cell) in immunofluorescence positive cells over the total number of cells in the same field. A minimum of 300 cells from randomly selected fields were scored.

### Detection of ROS production by DCF-DA

Intracellular ROS generation was measured by DCF-DA fluorescence probe using flow cytometer. Briefly, A549 and H358 cells (1 × 10^5^ cells/well) were seeded in a six-well plate, and different concentrations of deltarasin were added into the wells. After treatment with deltarasin for 24 h, the treated cells were detached with trypsin, and washed twice with PBS and incubated with 10 μM DCF-DA for 30 min at 37°C in the dark, and the fluorescence-stained cells were then analyzed using a FACS BD Aria III flow cytometer.

### Immunofluorescence

Cells were plated on six-well plates and grown overnight, then treated with 5 μM deltarasin or 5 mM 3-MA for 24 h. Cells were fixed with 4% paraformaldehyde for 20 min at 4° C, followed by permeabilized with 0.2% Triton X–100 in PBS for 10 min. Subsequently, the cells were blocked with 2% BSA/PBS for 30 min at room temperature, then incubated with Kras antibody (1:100) overnight at 4° C, followed by the secondary antibody (1:500) for 1 h at room temperature. The nuclear was stained with 1 μg/ml Hoechst staining for 10 min in the dark. The cells were visualized using fluorescent microscopy.

### RAS activation assay and immunoblotting

A549 cells were treated with deltarasin for 24 h at 5 μM. Cells were lysed in lysis buffer, and the volume of each sample was adjusted to 1 ml with 1× Assay Lysis Buffer. Then 40 μl of the Raf1 RBD Agarose bead slurry was swiftly added to each sample, and was incubated at 4° C for 1 h with gentle agitation, beads were washed three times with cold lysis buffer, and bounded protein was resuspended in 40 μl of 2× reducing SDS–PAGE sample buffer and were heated at 100° C for 5 min. The level of total Ras was detected after SDS–PAGE followed by western blot.

### Immunoprecipitations

Cells were lysed in NETN buffer (50 mM Tris (pH 8.0), 150 mM NaCl, 1 mM EDTA, 1% Nonidet P-40, and protease inhibitor tablets from Roche). The cell lysates (500 μg of protein) were immunoprecipitated for 1 h at 4° C with anti-KRAS (1 μg), and 50 μl of a 50% slurry of protein G beads was added, followed by incubation at 4° C for 1 h. Beads were washed three times with 500 μl of lysis buffer, and eluted by boiling in 20 μl of 2× SDS loading buffer. Immunoprecipitates were subjected to immunoblotting with anti-PDEδ antibody.

### Western blot analysis

Cells were washed twice with cold PBS, and then lysed in RIPA lysis buffer containing protease and phosphatase inhibitors. Protein concentration of the cell lysate was measured by using the Bio-Rad protein assay kit (Bio-Rad, Philadelphia, PA, USA). After equalizing the protein concentrations of the samples, 5× laemmli buffer was added and boiled at 100° C for 5 min. Equal amounts of protein (20–40 μg per lane) were separated with a 10% SDS–PAGE gel, then the separated proteins were transferred to a nitrocellulose (NC) membrane, which was then exposed to 5% non-fat dry milk in TBS containing 0.1% Tween 20 (0.1% TBST) for 1 h at room temperature with constant agitation, followed by overnight incubation at 4° C with primary antibodies. After washing three times with TBST, the membranes were incubated with secondary rabbit or mouse fluorescent antibodies, then the signal intensity of the membranes was detected by an LI-COR Odessy Scanner (Belfast, ME, USA). All primary antibodies were diluted in 1:1000, while their recommended secondary antibodies were diluted in 1:10,000.

### Mouse xenograft assays

All animal experiments were performed in compliance with institutional animal care guidelines and according to committee-approved protocols. About 2.5 × 10^6^ A549 cells were mixed with free FBS medium in 2:1 ratio plus growth factor-reduced matrigel (Becton Dickinson), and a 150 μl mixture was implanted into the right flank of 6-week-old nude female mice by s.c. injection. Xenografts were allowed to grow to a size around 60 mm^3^, and five mice per group were treated with vehicle (2% (vol/vol) DMSO, 40% (vol/vol) PEG400, and 5% (vol/vol) Tween 80 in normal saline) or deltarasin (15 mg/kg) via intraperitoneal (ip) injection daily for 21 days. Body weights were recorded and monitored for any signs of toxicity. Tumors were measured every third day using calipers, and the volume was estimated using the following formula: Tumor volume (mm^3^) = length (mm) × width (mm)^2^ × π/6. Finally, tumors were excised and retained for further analysis.

### Histology and immunohistochemistry

Tissues were fixed in 10% formalin, embedded in paraffin, and sections (5 μm) were prepared. The sections were dewaxed in xylene, dehydrated using a series of alcohol gradations, and stained with hematoxylin and eosin (H&E) for histological analysis. For immunohistochemistry, antigen retrieval was performed using Novocastra Epitope Retrieval Solution (pH 6.0) in a PT Link Dako pre-treatment module at 97 °C for 20 min, then cool down to 65 °C, and the sections were then brought to room temperature and rinsed in PBS. After neutralization of the endogenous peroxidase with 1% H_2_O_2_ and a specific protein block, the sections were incubated overnight at 4 °C with the primary antibodies. After incubation for 45 min with a HRP-conjugated anti-rabbit secondary antibody, the sections were visualized with diaminobenzidine (DAB), counterstained with haematoxylin, dehydrated, cleared, and mounted with permount. The following antibodies were used: p-ERK, p-AKT, Ki-67, and cleaved caspase-3.

### Statistical analysis

Statistical analysis was conducted using Graph Prim5.0. One-way analysis of variance (ANOVA) or Student’s *t* test was used to assess significant differences between data sets. Values of <0.05 were considered as significant.
